# Hyperactivity of the default-mode network in first-episode, drug-naive schizophrenia at rest revealed by family-based case–control and traditional case–control designs

**DOI:** 10.1097/MD.0000000000006223

**Published:** 2017-03-31

**Authors:** Wenbin Guo, Feng Liu, Jindong Chen, Renrong Wu, Lehua Li, Zhikun Zhang, Huafu Chen, Jingping Zhao

**Affiliations:** a Department of Psychiatry, the Second Xiangya Hospital, Central South University, Changsha, Hunan; b Key Laboratory for NeuroInformation of Ministry of Education, School of Life Science and Technology, University of Electronic Science and Technology of China, Chengdu, Sichuan; c Mental Health Institute of the Second Xiangya Hospital, Central South University, Changsha, Hunan, China; d Mental Health Institute of the Second Xiangya Hospital, Central South University, Changsha, Hunan Sheng; e National Clinical Research Center on Mental Disorders, Changsha, Hunan, China; f National Technology Institute on Mental Disorders, Changsha, Hunan, China; g Hunan Key Laboratory of Psychiatry and Mental Health, Changsha, Hunan, China.

**Keywords:** default-mode network, family-based case–control design, fractional amplitude of low-frequency fluctuation, schizophrenia

## Abstract

Abnormal regional activity and functional connectivity of the default-mode network (DMN) have been reported in schizophrenia. However, previous studies may have been biased by unmatched case–control design. To limit such bias, the present study used both the family-based case–control design and the traditional case–control design to investigate abnormal regional activity of the DMN in patients with schizophrenia at rest.

Twenty-eight first-episode, drug-naive patients with schizophrenia, 28 age-, sex-matched unaffected siblings of the patients (family-based controls, FBC), and 40 healthy controls (HC) underwent resting-state functional magnetic resonance imaging (fMRI) scans. The group-independent component analysis and fractional amplitude of low-frequency fluctuation (fALFF) methods were used to analyze the data.

Patients with schizophrenia show increased fALFF in an overlapped region of the right superior medial prefrontal cortex (MPFC) relative to the FBC and the HC. Compared with the HC, the patients and the FBC exhibit increased fALFF in an overlapped region of the left posterior cingulate cortex/precuneus (PCC/PCu). Furthermore, the *z* values of the 2 overlapped regions can separate the patients from the FBC/HC, and separate the patients/FBC from the HC with relatively high sensitivity and specificity.

Both the family-based case–control and traditional case–control designs reveal hyperactivity of the DMN in first-episode, drug-naive patients with paranoid schizophrenia, which highlights the importance of the DMN in the neurobiology of schizophrenia. Family-based case–control design can limit the confounding effects of environmental factors in schizophrenia. Combination of the family-based case–control and traditional case–control designs may be a viable option for the neuroimaging studies.

## Abstract 2

Supplemental Digital Content is available in the text

## 1 Introduction

Schizophrenia is a devastating psychiatric disorder characterized by distorted thoughts, flat affect as well as cognitive deficits.^[1]^ Recent evidence from neuroimaging studies suggests that schizophrenia may be the result of aberrant brain connectivity at the network level.^[2]^ One of the most examined networks is the default-mode network (DMN), which repeatedly shows significant functional connectivity (FC) at rest and across a wide variety of tasks.^[3–5]^ The DMN includes brain regions such as the posterior cingulate cortex/precuneus (PCC/PCu), medial prefrontal cortex (MPFC), inferior parietal, and parahippocampal gyrus.^[3]^ This network has been indicated to act as a key role in self-referential and reflective activity^[4,6]^ as well as in attending to internal and external stimuli.^[3,6,7]^

Abnormal regional activity and FC of the DMN have been reported in schizophrenia, but the results are inconsistent: FC increase,^[8–14]^ FC decrease,^[15–18]^ or both.^[19,20]^ One study even reported no FC difference within the DMN between patients with schizophrenia and controls.^[21]^ Recently, we applied a network homogeneity (NH) method to examine the NH of the DMN in patients with schizophrenia and their unaffected siblings.^[22,23]^ The findings exhibited that patients with schizophrenia showed increased and decreased NH in certain brain regions of the DMN, whereas the unaffected siblings exhibited decreased NH in certain brain regions of the DMN. Similar phenomenon is revealed in schizophrenia in regard to regional activity of the DMN. For example, both increased and decreased regional activities in the MPFC have been reported in schizophrenia with the amplitude of low-frequency fluctuation (ALFF) method.^[24,25]^

The inconsistent findings of abnormal regional activity and FC of the DMN in schizophrenia may result from many factors, such as sample heterogeneity, sample size, scanners, analysis methods, long illness duration, and potential medication effects. Particularly, most of the above-mentioned studies apply a traditional case–control design. Traditional case–control design is useful to identify differences between patients with schizophrenia and controls. However, the traditional case–control design may be confounded by the effects of environmental factors in schizophrenia, and possibly acquires false-positive results, though the controls and the patients are from the same geographic area. By contrast, family-based case–control design is powerful to reduce the confounding effects caused by environmental factors in the studies of gene-related illness such as schizophrenia. The efficiency to distinguish genetic effects is obtained at the cost of overmatching on environmental factors.^[26]^ Furthermore, it is important to conduct studies in first-episode, drug-naive patients with schizophrenia to limit the potential effects caused by medication and long illness duration, which seem to have an effect on gray matter volume and FC in schizophrenia.^[27,28]^

Here, we used a combination of the family-based case–control design and traditional case–control design to explore regional activity of the DMN in first-episode, drug-naive patients with schizophrenia. The unaffected siblings of the patients were recruited as the family-based controls (FBC). To reduce the illness heterogeneity, we recruited only patients with paranoid schizophrenia, though the term “paranoid schizophrenia” was no longer used in the new DSM-V system. The fractional ALFF (fALFF) method was applied to examine regional activity of the DMN in schizophrenia. The fALFF method provides a specific index of low-frequency oscillatory phenomena.^[29]^ This method has been well applied in schizophrenia.^[24,30]^ Given that increased and decreased FC and regional activity in brain regions of the DMN were reported in schizophrenia, we hypothesized that patients with schizophrenia would exhibit abnormal regional activity of the DMN compared with the controls. We also examined the correlations between abnormal regional activity of the DMN and symptom severity because such correlations have been reported in schizophrenia.^[16]^

## 2 Methods

### 2.1 Participants

This study was executed in accordance with the Helsinki Declaration.^[31]^ Thirty right-handed patients with schizophrenia were recruited from Mental Health Center, the First Affiliated Hospital of Guangxi Medical University in China. The diagnosis of schizophrenia was confirmed on the basis of the Structured Clinical Interview of the DSM-IV (SCID).^[32]^ To limit symptom heterogeneity, we recruited only patients with paranoid schizophrenia. The patients aged from 18 to 30 years and had more than 9 years of education. They were drug-naive and at their first episode with less than 3 years of illness duration. The exclusion criteria were neurological disorders, severe medical disorders, substance abuse, or electroconvulsive therapy. Positive and Negative Symptom Scale (PANSS) was used to assess symptom severity.

The right-handed FBC came from the same families of the patients. Each patient was matched with a family-based control. Forty-two right-handed healthy controls (HC) were recruited from the community. The controls were checked with SCID, nonpatient edition.^[32]^ They were 18 to 30 years old with more than 9 years of education. The exclusion criteria were any neurological disorders, severe medical disorders, substance abuse, or psychiatric disorders. Potential HC with a first-degree relative having psychiatric disorders was also excluded.

Each participant gave his/her written informed consent. The study was approved by the local ethics committee of the First Affiliated Hospital of Guangxi Medical University.

### 2.2 Data acquisition and preprocessing

A Siemens (Trio) 3T scanner was applied to acquire MRI images. The images were processed with the Data Processing Assistant for Resting-State fMRI (DPARSF) software.^[33]^ Details of image acquisition and preprocessing could be found in the Supplementary files.

### 2.3 DMN identification

Group-independent component analysis (ICA) was used to select the DMN mask from all participants as suggested by 2 recent studies.^[30,34]^ Three steps, including data reduction, independent component separation, and back reconstruction, were conducted with the toolbox GIFT (http://mialab.mrn.org/software/#gica). The DMN mask (Figure S1) was selected on the basis of the templates provided by GIFT.

### 2.4 fALFF analysis

According to a previous study,^[29]^ fALFF was computed as the ratio of power spectrum of low-frequency to that of the entire frequency range with the DPARSF software. First, the time series of each voxel was transferred to the frequency domain without band-pass filtering with a Fast Fourier Transform and the power spectrum was acquired. Then, the square root was calculated at each frequency of the power spectrum and the mean square root was obtained across 0.01 to 0.08 Hz at each voxel. The sum of amplitudes across 0.01 to 0.08 Hz was divided by that across the entire frequency range. Finally, the fALFF of each voxel was divided by the whole mean fALFF value within a mask for standardization purpose.^[35]^

### 2.5 Statistical analysis

When appropriate, demographical data were compared with Chi-square test and analysis of variance (ANOVA). The comparisons of fALFF within the DMN were compared with analysis of covariance (ANOVA) based on the general linear model (GLM), followed by post hoc *t* tests (paired-sample *t* tests were applied to compare group differences between the patients and the FBC). Age and education level were applied as covariates to limit the potential effects of these variables. The framewise displacement (FD) values were calculated for each participant and also applied as a covariate. The significance level was set at *P* < 0.005 for multiple comparisons corrected by Gaussian Random Field (GRF) theory (voxel significance: *P* < 0.001, cluster significance: *P* < 0.005).

To decide the overlap between the results from the family-based case–control design and the traditional case–control design, brain regions with abnormal fALFF were overlaid on the same template. Mean *z* values in the overlapped regions were extracted for further receiver operating characteristic (ROC) curves analyses.

Brain regions with abnormal fALFF were set as regions-of-interest (ROIs). Mean fALFF values were extracted for linear correlation analyses between these abnormal values and the PANSS scores or the illness duration in the patients. The results were Bonferroni corrected at *P* < 0.05.

## 3 Results

### 3.1 Demographics and clinical characteristics

One patient, 1 family-based control, and 2 HC are excluded from the analysis due to excessive head motion. Therefore, the final analysis includes 28 patient–FBC pairs and 40 HC. The 3 groups have no significant differences in age, sex ratio, years of education, and FD values (Table 1). The patients achieve a mean PANSS total score of 88 with the illness duration around 24 months.

**Table 1:**
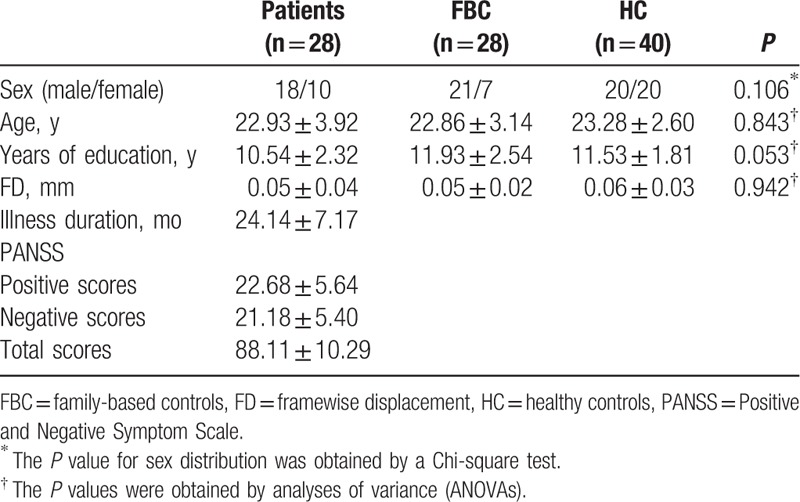
Characteristics of the participants.

### 3.2 Group differences in fALFF

**Figure 1:**
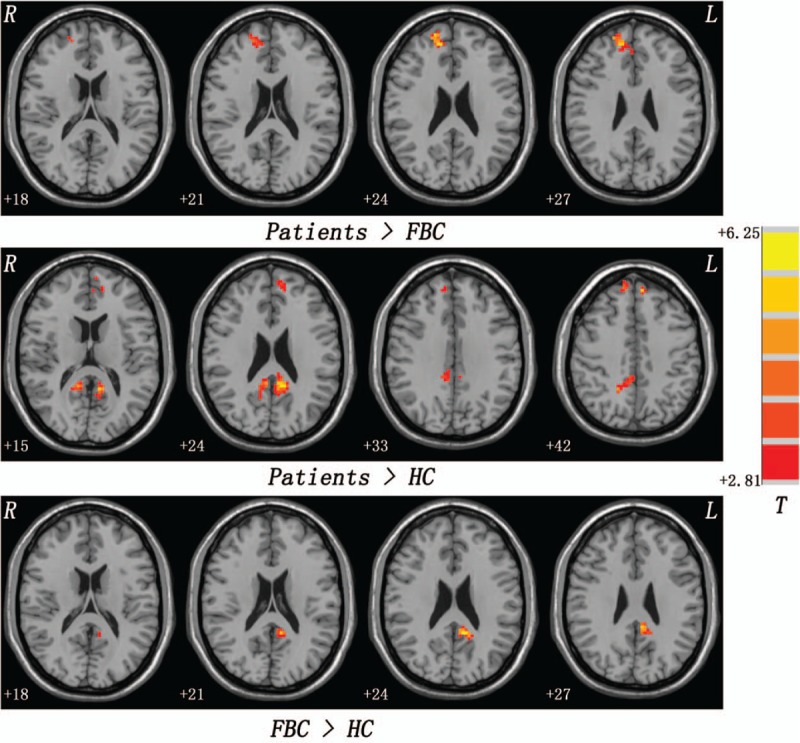
Group differences in fALFF. Red denotes increased fALFF. fALFF = fractional amplitude of low-frequency fluctuation.

As shown in Figure S2, ANCOVA reveals that main effects of groups in fALFF exist in brain regions of the DMN, such as the bilateral superior MPFC and bilateral PCC/PCu. Compared with the FBC, patients with schizophrenia show increased fALFF in the right superior MPFC. The patients exhibit increased fALFF in the bilateral superior MPFC and bilateral PCC/PCu relative to the HC. Compared with the HC, the FBC have increased fALFF in the left PCC/PCu (Fig. 1 and Table 2). No significantly decreased fALFF is found in the patients relative to the controls.

**Table 2:**
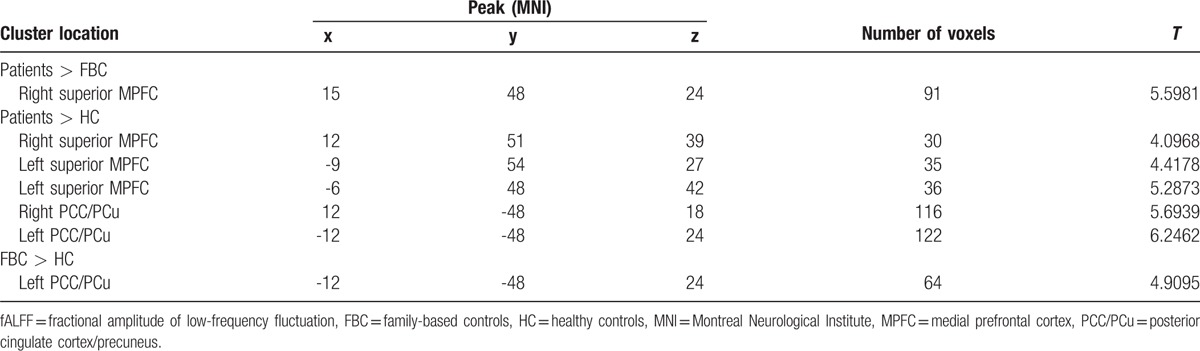
Regions with increased fALFF in the patients/FBC.

### 3.3 Overlap of the fALFF results

**Figure 2:**
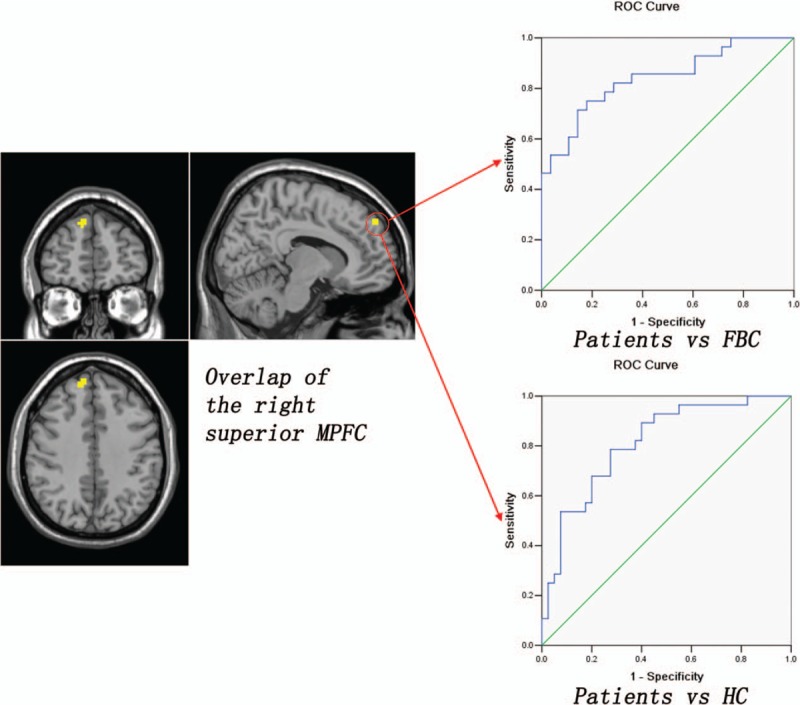
Overlap of the right superior MPFC that could be used to separate the patients from the FBC/HC with relatively high sensitivity and specificity. FBC = family-based controls, HC = healthy controls, MPFC = medial prefrontal cortex.

**Figure 3:**
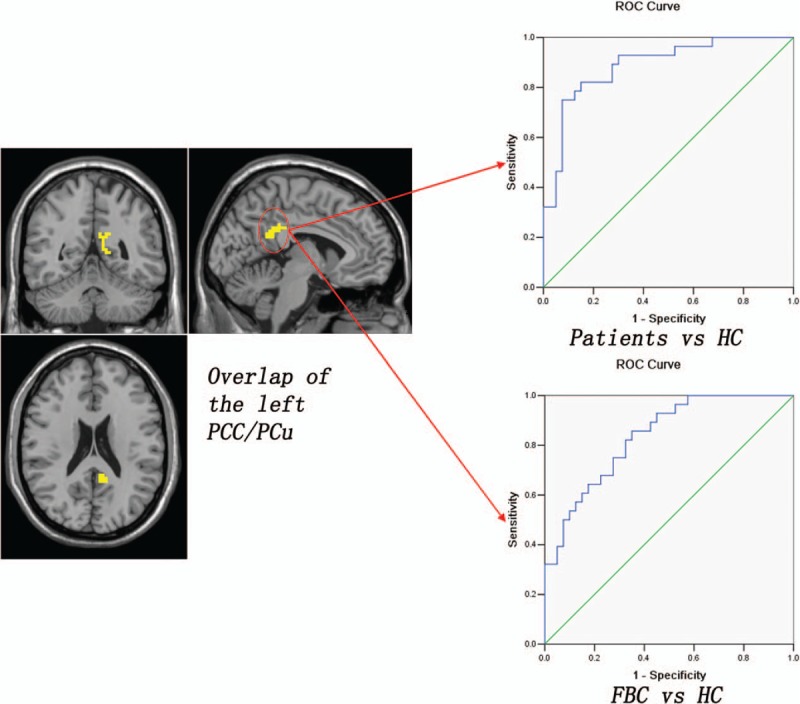
Overlap of the left PCC/PCu that could be used to separate the patients/FBC from the HC with relatively high sensitivity and specificity. FBC = family-based controls, HC = healthy controls, PCC/PCu = posterior cingulate cortex/precuneus.

To decide the overlap between the results from the family-based case–control design and the traditional case–control design, the fALFF results are overlaid on the same template. Patients with schizophrenia show increased fALFF in an overlapped region of the right superior MPFC (13 voxels) relative to the FBC and the HC (Fig. 2). Compared with the HC, both the patients and the FBC exhibit increased fALFF in an overlapped region of the left PCC/PCu (51 voxels) (Fig. 3). Further ROC analyses show that the *z* values of the 2 overlapped regions can separate the patients from the FBC/HC, and separate the patients/FBC from the HC with relatively high sensitivity and specificity (Figs. 2 and 3 and Table 3).

**Table 3:**
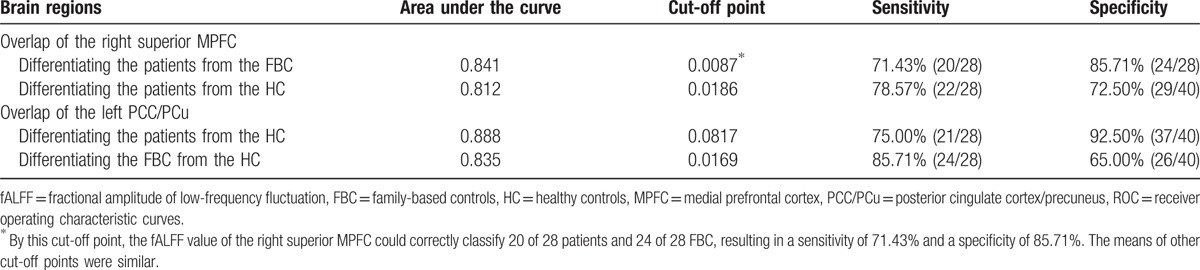
ROC analysis for differentiating the patients/FBC from the FBC/HC.

### 3.4 Correlations between increased fALFF and clinical variables

No correlations are found between the increased fALFF values and clinical variables in the patients. There are also no correlations between the increased fALFF values and age or years of education.

## 4 Discussion

Using the family-based case–control design and the traditional case–control design, this study reveals increased regional activity of the DMN in the patients. Patients with schizophrenia show increased fALFF in an overlapped region of the right superior MPFC relative to the FBC and the HC. Compared with the HC, the patients and the FBC exhibit increased fALFF in an overlapped region of the left PCC/PCu. Furthermore, the *z* values of the 2 overlapped regions can separate the patients from the FBC/HC, and separate the patients/FBC from the HC with relatively high sensitivity and specificity.

There are 2 novel aspects in the present study. First, the application of the family-based case–control design and the traditional case–control design is novel. Schizophrenia is a complex disease resulting from several genes with small effects that interact with environmental risk factors.^[31]^ Even though the patients and the controls come from the same large geographic area, the traditional case–control design cannot exclude the confounding effects from the environmental factors and possibly acquires false-positive results. By contrast, family-based case–control design can limit the effects caused by unmatched cases and controls. The patients and the controls are well matched on many genetic and ethnic confounding factors. Therefore, family-based case–control design enhances the specificity of the findings for schizophrenia. In the present study, the traditional case–control results of increased fALFF in the left superior MPFC and right PCC/PCu may be due to the genetic and ethnic confounding factors compared with the family-based case–control results. However, the efficiency of the family-based case–control design is acquired at the cost of overmatching the genetic and ethnic factors.^[26]^ Previous studies have shown that patients with schizophrenia and FBC share abnormal activity and connectivity in certain brain regions.^[18,36,37]^ When the family-based case–control design is used, the shared abnormalities may be overmatched. For example, the patients and the FBC share increased fALFF in the left PCC/PCu relative to the HC in the present study, which may be a trait alteration for schizophrenia. This trait alteration is overmatched by the family-based case–control design and disappears in the comparisons between the patients and the FBC. Furthermore, there are overlaps between the family-based case–control results and the traditional case–control results, which can be applied to discriminate the patients/FBC from the FBC/HC. The overlaps indicate that the 2 designs are complementary and reliable in the present study.

The second novel aspect of this study is hyperactivity of the DMN in schizophrenia. According to the “disconnection” hypothesis, the patients are expected to show decreased regional activity and connectivity of the DMN. Inconsistent with the “disconnection” hypothesis, the present patients show hyperactivity of the DMN. The prevailing “disconnection” hypothesis is based on studies with chronic and/or medicated schizophrenia.^[38]^ By contrast, study with early-course, drug-naive schizophrenia has reported increased frontal connectivity.^[39]^ In our previous study,^[36]^ patients with first-episode, drug-naive schizophrenia also showed increased cerebellar-DMN connectivity. Combined with the findings from chronic schizophrenia, it may be that the DMN has hyperactivity around the illness onset, and it exhibits a declining regional activity over the illness duration that may result in persistent disability and treatment resistance observed in some chronic schizophrenia.^[38]^ Hyperactivity of the DMN is often explained as a compensatory effort^[40–42]^ that may be modulated by inflammation process in the early course of the disease. In that stage, proinflammatory cytokines (such as interleukin-6) can activate the astrocytes that show increased metabolism and blood flow (hyperfunction).^[43]^ Regional hyperfunction can contribute to increased regional activity and FC of the network.^[36]^ Hence, it is no wonder for the present patients to show hyperactivity of the DMN.

Several limitations should be addressed when interpreting the present results. First, the present study focuses on the DMN. It enhances the specificity of the findings from the DMN. For the same reason, the results from other brain regions have been neglected. Second, a trend level of significance of group differences in years of education is observed in the present study. It is understandable that the patients receive less years of education than the controls. The effects of unmatched education level may not be completely eliminated, although this variable is used as a covariate in the group comparisons. Finally, the sample size is relatively small. Large sample size is needed to elucidate the subtle changes of brain activity and FC in schizophrenia.

Despite the limitations, the present study observes hyperactivity of the DMN in first-episode, drug-naive patients with paranoid schizophrenia revealed by the family-based case–control and traditional case–control designs, which highlights the importance of the DMN in the neurobiology of schizophrenia. Family-based case–control design can limit the confounding effects of environmental factors in schizophrenia. Combination of the family-based case–control and traditional case–control designs may be a viable option for the neuroimaging studies to examine the abnormalities specified to the disease in schizophrenia and the trait alterations shared by the patients and the FBC.

## Supplementary Material

Supplemental Digital Content
