# Supplementary material for: Hyperactivity of the default-mode network in first-episode, drug-naive schizophrenia at rest revealed by family-based case–control and traditional case–control designs
Source: Medicine (Baltimore). 2017 Mar 31;96(13):e6223. doi: 10.1097/MD.0000000000006223 (PMC5380243; doi:10.1097/MD.0000000000006223)
Supplement: Supplemental Digital Content [file medi-96-e6223-s001.doc]

**Title:**

**Hyperactivity of the default-mode network in first-episode, drug-naive schizophrenia at rest revealed by family-based case-control and traditional case-control designs**

**Authors:**

Wenbin Guo, MD; Feng Liu, PhD; Jindong Chen, MD; Renrong Wu, MD; Lehua Li, MD; Zhikun Zhang, MD; Huafu Chen, PhD; Jingping Zhao, MD

**Affiliation/address:**

From Department of Psychiatry, the Second Xiangya Hospital, Central South University, Changsha, Hunan 410011, China (GW, CJ, WR, LL, ZJ); Key Laboratory for NeuroInformation of Ministry of Education, School of Life Science and Technology, University of Electronic Science and Technology of China, Chengdu, Sichuan, China (LF, CH); Mental Health Center, the First Affiliated Hospital, Guangxi Medical University, Nanning, Guangxi 530021, China (ZZ); Mental Health Institute of the Second Xiangya Hospital, Central South University, China (GW, CJ, WR, LL, ZJ); National Clinical Research Center on Mental Disorders, China (GW, CJ, WR, LL, ZJ); National Technology Institute on Mental Disorders, China (GW, CJ, WR, LL, ZJ); Hunan Key Laboratory of Psychiatry and Mental Health, China (GW, CJ, WR, LL, ZJ).

**Corresponding author:**

Wenbin Guo

Mental Health Institute of the Second Xiangya Hospital, Central South University, The China National Clinical Research Center for Mental Health Disorders，National Technology Institute of Psychiatry, Key Laboratory of Psychiatry and Mental Health of Hunan Province, 139 Middle Renmin Road, Changsha, Hunan 410011, People’s Republic of China.

E-mail: guowenbin76@163.com

Tel.: +86 731 85360921

*Data acquisition and preprocessing*

MRI images were obtained on a Siemens 3T scanner. The participants were required to remain motionless and awake with their eyes closed. Soft earplugs and foam pads were used to decrease scanner noise and head motion. Resting-state functional images were obtained with a gradient-echo echo-planar imaging (EPI) sequence using the following parameters: repetition time/echo time = 2000 ms/30 ms, 30 slices, 64 × 64 matrix, 90° flip angle, 24 cm field of view, 4 mm slice thickness, 0.4 mm gap, and 250 volumes lasting for 500 s. High-resolution T1-weighted images were also obtained with a three-dimensional spoiled gradient-recalled sequence in an axial orientation: repetition time = 8.5 ms, echo time = 2.98 ms, inversion time = 900 ms, flip angle = 9°, acquisition matrix = 256 × 256, field of view = 240 mm × 240 mm, slice thickness = 1 mm, no gap, and 176 slices.

Data Processing Assistant for Resting-State fMRI (DPARSF) software was applied to preprocess the images. After slice timing and head motion correction, participants with more than 2 mm of maximal translation and 2° of maximal rotation were excluded. To correct the inhomogeneous B0 field induced distortion, we performed the following steps . First, the T1 images were reoriented and coregistered to the mean functional images. Subsequently, the T1 images were segmented into gray matter, white matter, and cerebrospinal fluid by using a unified segmentation algorithm . High-dimensional normalization by the diffeomorphic anatomical registration through exponentiated lie algebra method (DARTEL) was applied to register the segmented images to the Montreal Neurological Institute (MNI) space. Tissue deformation was used to modulate the segmented gray matter images, and the normalized and modulated volumes (voxel size, 1.5 × 1.5 × 1.5mm^3^) were smoothed with an 8 mm full-width at half-maximum Gaussian kernel. Next, the motion-corrected functional volumes were spatially normalized to the MNI space and resampled to 3 × 3 × 3 mm^3^ voxels using the normalization parameters estimated during unified segmentation. After normalization, the images were smoothed (with an 8 mm full width at half maximum Gaussian kernel), bandpass filtered (0.01 - 0.08 Hz), and linearly detrended. Several covariates, including Friston-24 head motion parameters acquired by rigid body correction, signal from a ventricular region of interest (ROI), and signal from a region centered in the white matter, were removed. The global signal was not removed as indicated in a previous study .

**References**

1. Yan C, Zang Y. DPARSF: A MATLAB toolbox for "pipeline" data analysis of resting-state fMRI. Front Syst Neurosci2010;4:13.

2. Kybic J, Thevenaz P, Nirkko A, Unser M. Unwarping of unidirectionally distorted EPI images. IEEE Trans Med Imaging2000 Feb;19(2):80-93.

3. Ashburner J, Friston KJ. Unified segmentation. Neuroimage2005 Jul 1;26(3):839-51.

4. Hahamy A, Calhoun V, Pearlson G, Harel M, Stern N, Attar F *et al.* Save the global: global signal connectivity as a tool for studying clinical populations with functional magnetic resonance imaging. Brain Connect2014 Aug;4(6):395-403.


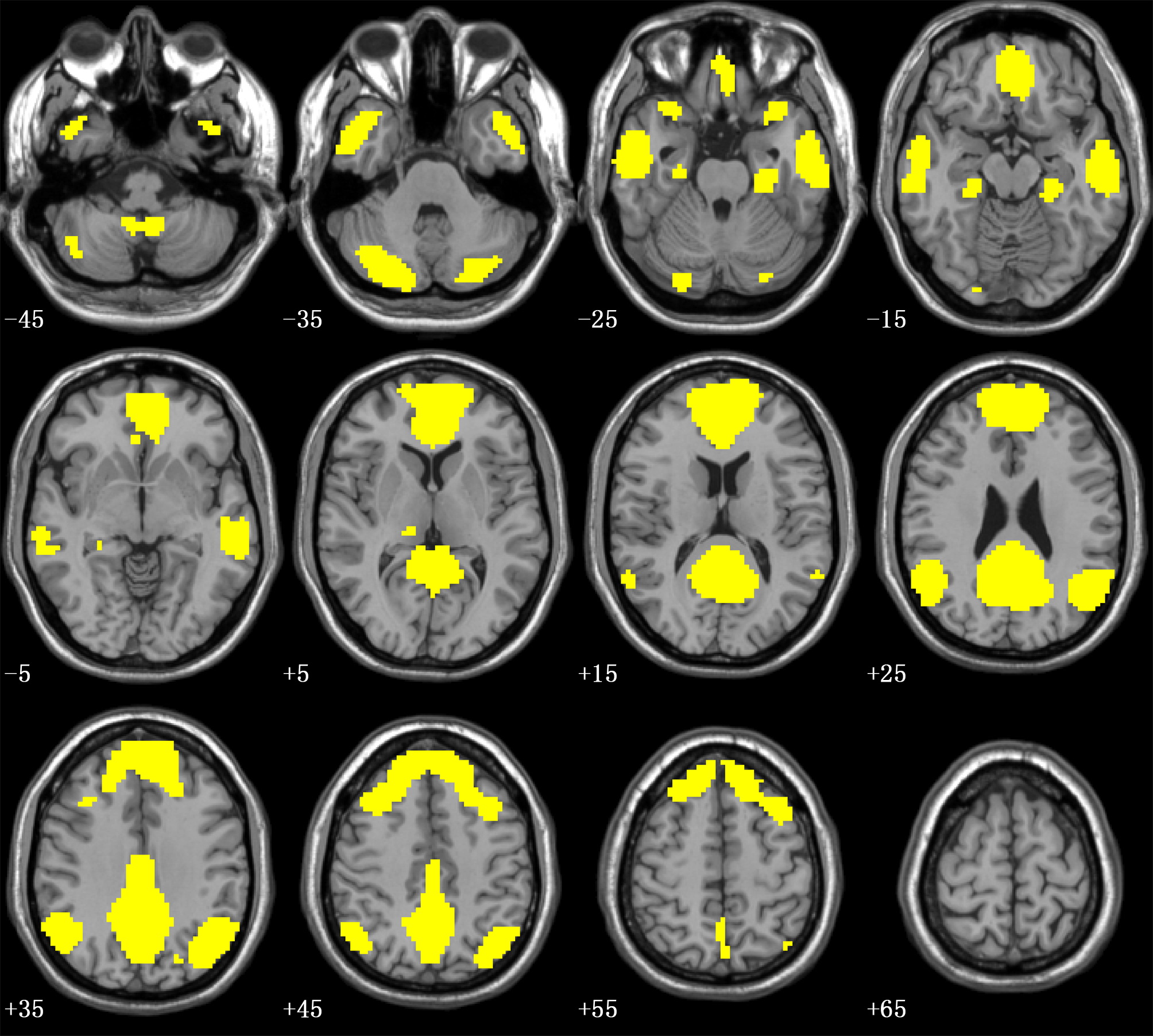


Figure S1. DMN mask generated from all participants by group independent component analysis. DMN = default-mode network.


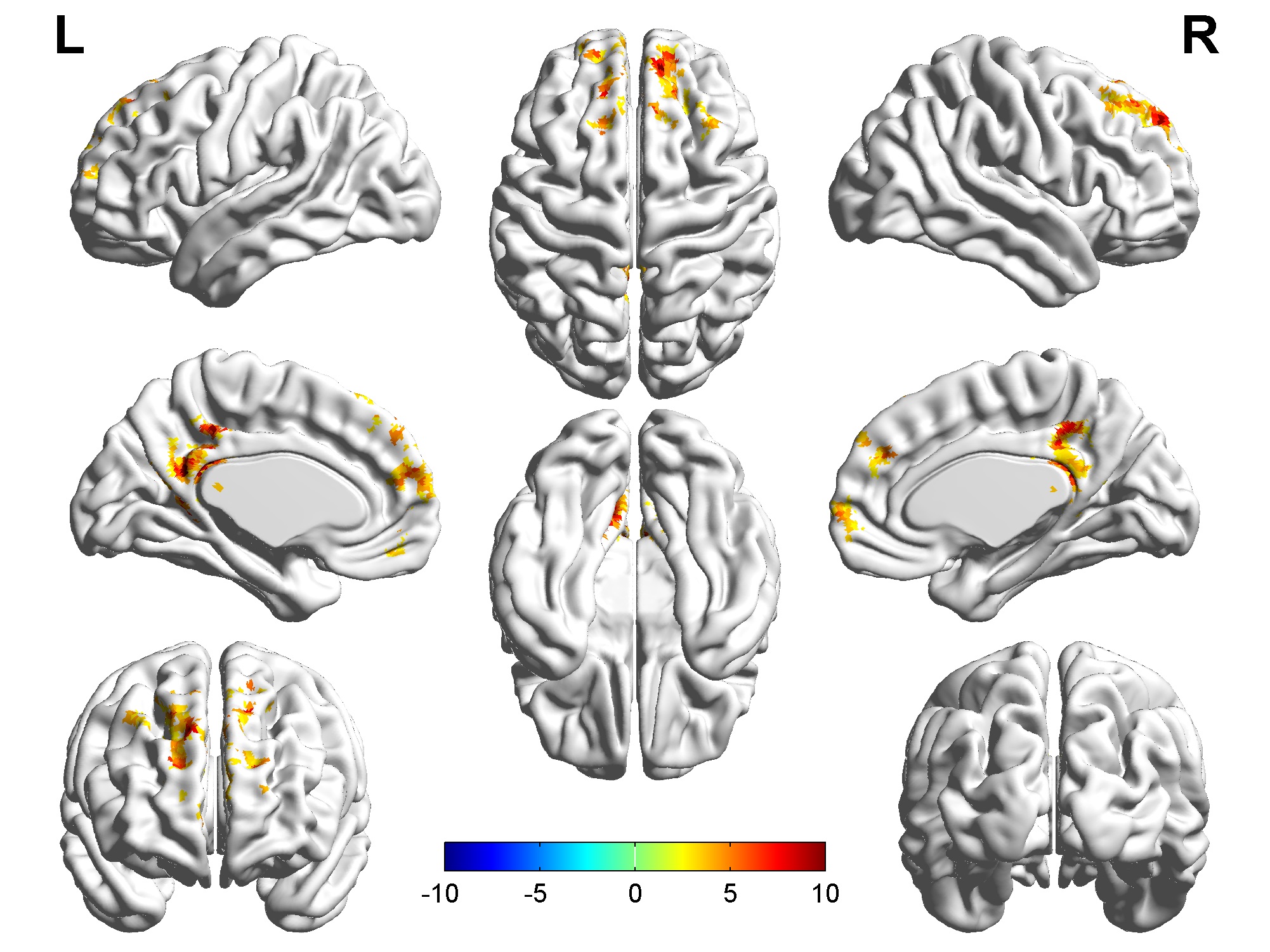


Figure S2. Brain regions of the DMN showing main effects of groups in fALFF by using ANCOVA. DMN = default-mode network; fALFF = fractional amplitude of low-frequency fluctuation; ANCOVA = analyses of covariance.
